# Endotoxemia, vitamin D and premature biological ageing in Arab adults with different metabolic states

**DOI:** 10.1016/j.sjbs.2022.03.026

**Published:** 2022-03-31

**Authors:** Nasser M. Al-Daghri, Shaun Sabico, Mohammed G.A. Ansari, Saba Abdi, Gyanendra Tripathi, George P. Chrousos, Philip G. McTernan

**Affiliations:** a Biochemistry Department, Chair for Biomarkers of Chronic Diseases, King Saud University, Riyadh 11451, Saudi Arabia; b Human Sciences Research Centre, School of Human Sciences, University of Derby, Derby, DE122 1GB, UK; c University Research Institute of Maternal and Child Health and Precision Medicine, UNESCO Chair on Adolescent Health Care, National and Kapodistrian University of Athens, 11527 Athens, Greece, Greece; d Department of Biosciences, School of Science and Technology, Nottingham Trent University, Nottingham, NG1 8NS, UK

**Keywords:** Telomere length, Endotoxin, Endotoxin/HDL-Cholesterol ratio, Systemic inflammation, Oxidative stress, Age

## Abstract

There are limited studies on the association of endotoxin, a potent mediator of gut-derived inflammation and telomere length (TL). We investigated (1) the influence of adiposity on endotoxin and TL amongst Saudi adults according to type 2 diabetes mellitus (T2DM) status and (2) the influence vitamin D may have on TL attrition. Anthropometric data and fasting blood samples were taken from 775 Saudi adults visiting different primary care centers in Riyadh [387 T2DM and 388 non-T2DM]. TL, derived from peripheral blood mononuclear cells, was analyzed by Quantitative real-time polymerase chain reaction and circulating endotoxin levels by Limulus Amebocyte Lysate assay. Subjects were stratified based on obesity and T2DM status. A significant lower TL was observed in the non-obese T2DM group as compared with their non-obese, non-T2DM counterparts (p = 0.002). Significant inverse associations between TL, endotoxin and endotoxin activity were observed in the cohort with obesity. Regression analysis showed that endotoxin was a significant predictor for TL in all subjects and even after stratification according to subgroups; with variances perceived in circulating TL stronger among non-T2DM obese (10%; p = 0.003) than non-T2DM non-obese (12%; p = 0.007). Also, in the non-T2DM group, TL and HDL-cholesterol predicted 29% of the variances perceived in 25(OH)D (p < 0.001). Taken together these findings show that circulating endotoxin and 25(OH)D are associated with premature biological ageing influenced by adiposity and metabolic state; suggesting future intervention studies to manipulate gut microbiome and or vitamin D levels may offer ways to mitigate premature TL attrition.

## 1 Introduction

Epidemiological investigations have shown that associations exist between diminished telomere length (TL) and a number of adverse health outcomes including risk of cardiovascular diseases, type 2 diabetes mellitus (T2DM) and cancer (Hoffmann and Spyridopoulos, 2011, Ma et al., 2011). In addition, many studies have investigated the relevance of chronic inflammation and oxidative stress as a pathophysiological basis of these adverse health outcomes (Ikeoka et al., 2010, Kamp and Weitzman, 2011). T2DM, in addition to other such metabolic diseases, represent a complex pathophysiological condition where molecular insight has sought to consider the role of inflammation. In the Arab setting, our previous studies on the associations between TL, and insulin resistance (IR) revealed an inverse relationship in both young and middle-aged cohorts (Al-Attas et al., 2010a, Al-Attas et al., 2010b); however, limited data has been documented on the relationship of TL with inflammation.

TL is critical for cell division as well as cell turnover. In most somatic cells, the progressive telomere shortening occurs as a normal process; however, a compensatory mechanism to add back “TTAGGG” repeats by enzyme telomerase occurs in hematopoietic stem cells and germ-line cells (Cong et al., 2002; Saretzki, 2018). Although telomere shortening can be observed with progressive cell division in vitro and as a natural part of ageing *in vivo* (Lindsey et al., 1991, Eisenberg, 2011), both intrinsic and extrinsic factors such as genetics, lifestyle, diet, and environmental factors can also play a role in the rate of shortening of TL (Falus et al., 2010, Starkweather et al., 2014). Interestingly, the non-modifiable and modifiable factors mentioned also place significant influence on age-associated chronic conditions including, T2DM, cancer and coronary heart disease (CHD) (Korat et al., 2014, Yu et al., 2016).

A systemic low-grade sub-clinical inflammatory state comprising the pathogenesis of T2DM has been proposed to arise through an imbalance in the production of pro- and anti-inflammatory adipocytokines, as well as the considered anti-inflammatory properties of vitamin D (Al-Daghri et al., 2017, Ansari et al., 2020). Adipose tissue also represents a site of an acute phase response and a considered site able to sequester circulating vitamin D (Wellen and Hotamisligil, 2005, Wozniak et al., 2009). Based on this knowledge there is a growing interest in studying the mediators of acute and chronic very low-grade inflammatory condition (Wani et al., 2021) and one such mediator appears to arise from the gut-derived lipopolysaccharide or endotoxin from the Gram-negative bacteria (Creely et al., 2007). Studies on age-associated diseases reveal the importance of a leaky gut resulting in the translocation of endotoxin into the bloodstream referred to as intestinal end toxemia (Munford, 2016) and its resulting effects on inflammation directly on human adipose tissue (Creely et al., 2007).

In addition to adipose tissue all nucleated human cells have the capacity to respond to endotoxin as part of their innate immune response where the myeloid differentiation factor 2/toll-like receptor 4 (MD2-TLR4) can sense circulating endotoxin and initiate a local or systemic inflammatory processes involved in pathologies as diverse as metabolic syndrome, atherosclerosis, obesity, and T2DM (Shimazu et al., 1999, Andreasen et al., 2008). Our previous studies have revealed that circulating levels of endotoxin, considered as an important mediator in triggering the inflammatory cascade, is elevated in subjects with T2DM, fatty liver disease, cardiovascular disease compared with their healthy counterparts (Al-Disi et al., 2015); and since inflammation can initiate genomic trauma at telomeric sites, it’s noteworthy to study the relationship of endotoxin with TL and the clinical implications of such association. Whilst there are previous studies examining the association of plasma inflammatory markers and TL (O'Donovan et al., 2011, Masi et al., 2012), the association between gut derived circulating endotoxin and TL has received little or no attention to date. Thus, this current study sought to investigate the association of endotoxin with TL attrition and vitamin D amongst adults with varying levels of adiposity and insulin resistance.

## 2 Materials and methods

### 2.1 Subjects, anthropometrics, blood samples and the study groups

Saudi Arabian subjects (n = 775, 374 men and 401 women) aged between 40 and 80 years were selected from various primary care centers around Riyadh from March 2014 to May 2016. The inclusion criteria were Saudi Arabian adults 40 years old and above, with or without T2DM. T2DM subjects were known cases without complications. Exclusion criteria were anyone with chronic conditions such as, liver, lung, kidney diseases and pregnant women and performed the experiment under the IRB no.8-25-454239 approval. Parameters such as, body weight (kg), height (cm), hip (cm), and blood pressure (mmHg) were analyzed. Body mass index (BMI) of all subjects was calculated (kg/m^2^). Fasting venous blood samples were extracted from each subject, processed, transported and stored immediately at the CBCD laboratory under appropriate conditions.

### 2.2 TL measurement

To isolate DNA from peripheral blood mononuclear cells (PBMC), genomic prep mini spin kit (GE healthcare, NJ, USA) was used as per the manufacturer’s protocol. DNA was extracted and resuspended in 200 μL of Tris-EDTA (TE buffer) and the amount of DNA was estimated using Nano-drop ND 1000 spectrophotometer (Nanodrop technologies, Wilmington, DE, USA) and purity (260/280 nm) was checked before further analysis. The RT PCR has been performed to determine the expression pattern of the particular gene using machine (Bio-Rad Laboratories, Hercules, CA, USA) was utilized to measure TL as described previously (Al-Attas et al., 2012). This experiment analyzes the abundance of DNA of telomere to a control gene that is non-variable in copy number (glyceraldehyde 3-phosphate dehydrogenase, GADPH). The amount of telomere sequence and single-copy gene are directly proportional to the cycle-threshold (Ct value) using forward and reverse primers were applied.

a) GADPH gene-

Forward: 5′ AACCAGCCAAGTACGATGACAT 3′.

Reverse: 5′ CCATCAGCAGCAGCCTTCA 3′.

b) Telomere primers-

Forward: Tel1b 5′ CGGTTTGTTTGGGTTTGGGTTTGGGTTTGGGTTTGGGTT 3′.

Reverse: Tel2b 5′ GGCTTGCCTACCCTTACCCTTACCCTTACCCTTACCCT 3′.

The PCR reactions were performed in triplicates in a 96-well PCR vial. Each plate also included two calibrators run in triplicate. The average inter-plate variation of the threshold cycle number between both primer pairs (ΔCT) across all plates was 5.04% and 5.11% for MRC5 and K1E72 respectively. Plots of normalized template quantity for standards versus ΔCT showed linear relations. The slopes of the graphs were used to convert the ΔCT for samples into initial amount of DNA and to transform the telomere/single copy gene SQ ratio to TL in base pairs (bp) (Cawthon, 2002, Yang et al., 2009).

### 2.3 Biochemical tests

Blood samples were collected from all subjects and serum samples were separated. Such samples were used for the determination of various biochemical parameters, including, lipid content, glycemic indices using insulin, fasting glucose, and 25(OH)D. Circulating triglycerides, HDL-cholesterol, total cholesterol and glucose were determined using the commercial kit (Konelab 20 Thermo-Fischer, Espoo, Finland). Fasting insulin levels were evaluated using a fluorescent microbead technology (Luminexcorp, Austin, Texas, USA). Serum 25(OH)D was evaluated using immunoassay method (IDS Ltd, Boldon Colliery, UK). All experiments were performed in triplicates and control experiments were performed to assure the quality of reproducible results (Sabico et al., 2019, Al-Daghri et al., 2021).

Serum endotoxin levels were determined as described previously with Limulus Amebocyte Lysate (LAL) assay kit (QCL 1000, Lonza, MD, USA) (Creely et al., 2007, Sabico et al., 2017, Sabico et al., 2019). The principle of the assay utilizes endotoxin present in the sample for the activation of a proenzyme in LAL which in-turn catalyzes the release of p-nitro aniline producing a yellow color, photo metrically estimated at 405 nm. A spike recovery was undertaken at a dilution of 1:40, which yielded a recovery of 60% which was within the acceptable range as per the manufacturer. The intra-assay and inter-assay variations validated for the kit were 3.9% and 9.6% respectively. The ratio of endotoxin and HDL-cholesterol (endotoxin/HDL-cholesterol) was used as a measure of endotoxin activity (Lassenius et al., 2011). HOMA-IR was determined from the sample as described previously by Bonora et al., 2002, and HOMA-β was calculated as described previously by Simental-Mendía et al. (2015).

### 2.4 Statistical analysis

SPSS V23 software was used for the analysis of data (SPSS, Chicago, IL). Kolmogorov-Smirnov test was performed and normality of the selected factors was tested. Additionally, *T*-test and Mann-Whitney *U* test were applied to reveal variations between normal and abnormal factors. Log-transformation was done to normalize non-normal continuous variables. Endotoxin and Endotoxin activity values were log-transformed after adding 1 (some of them yielded negative scores especially in non-T2DM group). Bivariate-associations were undertaken using Pearson test and presented as coefficients (r). Linear regression with all parameters assessed as independent variables.

## 3 Results

### 3.1 Body mass index analysis and determination of biochemical components

In this study, body mass index and biochemical characteristics of the individuals were presented (Table 1). The two groups differed in age, waist size and systolic blood pressure with T2DM group being older, and with a higher waist circumference and systolic blood pressure statistically significant compared to the non-T2DM group. As expected, subjects in T2DM group had higher circulating amounts of fasting glucose and triglyceride than their counterparts in non-T2DM group while vitamin D levels were significantly lower. The variation of total cholesterol, HDL-cholesterol and insulin were not statistically significant. Moreover, the calculated level of HOMA-IR and HOMA-β were statistically significant in T2DM group. The average level of endotoxin in human subjects with T2DM were statistically significant compared with the non-T2DM group (median level of 2.45 EU/mL in T2DM compared with 1.8 EU/mL in non-T2DM, p < 0.001); endotoxin activity was also higher in T2DM group. The average TL in T2DM group was 5.6 ± 1.3 Kbp, significantly less than in non-T2DM group (5.9 ± 1.4 Kbp, p = 0.001).

**Table 1:** Anthropometric characteristics, biochemical estimations, and TL of all subjects.

**Parameters**	**Non-T2DM (n = 388)**	**T2DM (n = 387)**	**P-value**
Age (years)	55.8 ± 7.6	58.7 ± 8.1	<0.001
BMI (Kg/m^2^)	30.9 ± 6.0	30.76 ± 5.7	0.76
BMI Category^* Normal Overweight Obese	53 (13.7) 124 (32.0) 191 (49.2)	54 (14.0) 129 (33.3) 196 (50.6)	0.99
Waist (cm)	98.8 ± 17.1	103.1 ± 13.3	0.001
Hips (cm)	105.9 ± 17.0	106.8 ± 13.4	0.42
Systolic BP (mmHg)	125.0 ± 15.6	132.56 ± 14.9	<0.001
Diastolic BP (mmHg)	78.2 ± 11.8	79.7 ± 12.9	0.13
Glucose (mmol/L)	5.7 ± 0.9	10.5 ± 3.7	<0.001
Insulin (μU/mL) #	11.2 (7.1,16.8)	10.1 (6.5,16.1)	0.11
HOMA-IR#	2.8 (1.6,4.6)	4.2 (2.7,7.6)	<0.001
HOMA-β#	111.7 (64.4,192.2)	32.0 (18.6,58.1)	<0.001
Total Cholesterol (mmol/L)	5.0 ± 1.1	5.1 ± 1.3	0.48
HDL-Chol (mmol/L)	0.99 ± 0.3	1.0 ± 0.3	0.78
Triglycerides (mmol/L) #	1.5 (1.1,2.1)	1.7 (1.3,2.3)	0.003
Endotoxin (EU/mL) #	1.8 (1.2,2.8)	2.4 (1.7,4.1)	<0.001
Endotoxin Activity#	1.7 (1.2,2.6)	2.8 (1.7,4.6)	<0.001
25(OH) D (nmol/L) #	52.1 (29.0,79.7)	41.0 (27.5,59.2)	<0.001
TL (Kbp)	5.9 ± 1.4	5.6 ± 1.3	0.001

**Note:** Normal continuous findings are described as mean ± standard deviation, whereas, non-normal continuous data (#) are described as median value (Q1, Q3) whilst categorical data (^) is presented as frequency (%).* represents that BMI data was missing for 20 non-T2DM and 8 T2DM subjects. P < 0.05 was considered as significant.

### 3.2 Clinical differences according to obesity

**Fig. 1:**
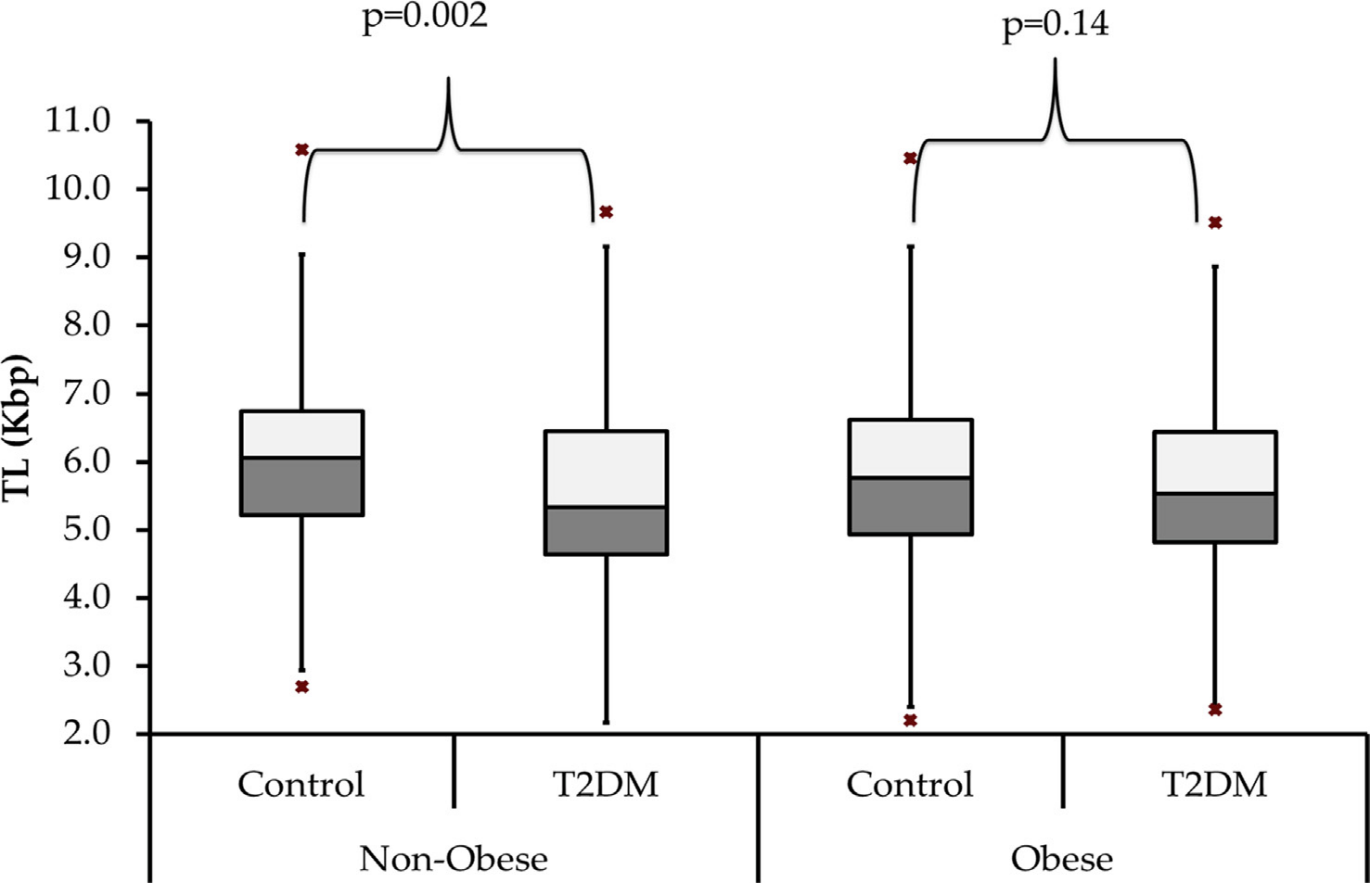
Mean TL in obese vs. non-obese groups according to T2DM status.

Data was stratified according to obesity and analyzed according to the study groups (Table 2). In the non-T2DM group, obese subjects had, as expected, significantly higher BMI, waist and hip circumferences (p-value: p < 0.001) as well insulin, HOMA-β and HOMA-IR than their non-obese counterparts. In the T2DM group, significant variations were determined between obese and non-obese, including significantly higher diastolic blood pressure (p = 0.02) and significantly younger age (p = 0.002) in the obese group as compared with the non-obese group. No differences in endotoxin, TL and 25(OHD) were noted. However, when subjects were grouped according to obesity status and stratified according to T2DM status, a significantly lower TL was observed in the non-obese T2DM group as compared with their non-obese, non-T2DM counterparts (p = 0.002) (Fig. 1).

**Table 2:** Differences in Clinical Characteristics of Studied Groups According to T2DM and Obesity Status.

**Parameters**	**Non-T2DM (n = 388)**			**T2DM (n = 387)**		
	**Non-Obese**	**Obese**	**P-Value**	**Non-Obese**	**Obese**	**P-Value**
N	180 (46.3)	208 (53.7)		185 (47.8)	202 (52.2)	
M/F	106/74	68/140		126/59	75/127	
Age (years)	55.9 ± 8.2	55.5 ± 7.0	0.61	60.1 ± 8.3	57.6 ± 7.5	0.002
BMI (Kg/m^2^)	26.0 ± 3.2	35.1 ± 4.4	<0.001	26.2 ± 2.4	35.0 ± 4.3	<0.001
Waist (cm)	91.5 ± 17.0	105.8 ± 14.3	<0.001	97.3 ± 11.3	108.3 ± 12.8	<0.001
Hips (cm)	98.1 ± 14.7	113.2 ± 15.9	<0.001	99.2 ± 10.9	113.6 ± 11.7	<0.001
Systolic BP (mmHg)	124.4 ± 15.5	125.4 ± 15.8	0.55	131.5 ± 14.1	133.4 ± 15.1	0.24
Diastolic BP (mmHg)	77.1 ± 10.1	79.4 ± 13.2	0.08	77.9 ± 11.0	81.2 ± 14.2	0.02
Glucose (mmol/L)	5.67 ± 0.9	5.66 ± 0.9	0.95	10.3 ± 3.6	10.7 ± 3.9	0.31
Insulin (μU/mL) #	10.4 (6.4–16.0)	12.7 (7.8–18.8)	0.01	8.9 (5.5–15.2)	10.9 (7.0–18.1)	0.01
HOMA-IR#	2.4 (1.5–4.2)	3.0 (1.9–5.2)	0.02	3.6 (2.4–7.5)	5.0 (3.0–7.8)	0.009
HOMA-β#	102.3 (62.6–163.5)	126.1 (71.7–211.7)	0.03	28.1 (17.5–53.4)	35.4 (21.3–60.8)	0.08
T. Cholesterol (mmol/L)	4.9 ± 1.1	5.0 ± 1.1	0.38	5.1 ± 1.2	5.1 ± 1.4	0.89
HDL-Chol (mmol/L)	1.0 ± 0.32	1.0 ± 0.31	0.94	1.0 ± 0.35	1.0 ± 0.32	0.83
LDL- Chol (mmol/L)	3.2 ± 1.0	3.23 ± 1.0	0.76	3.2 ± 1.0	3.2 ± 1.2	0.67
LDL/HDL ratio	3.8 ± 2.7	3.78 ± 2.8	0.96	3.5 ± 1.7	3.4 ± 1.4	0.38
Triglycerides (mmol/L) #	1.7 ± 1.0	1.8 ± 0.8	0.62	2.0 ± 1.2	2.0 ± 0.97	0.83
Endotoxin (EU/mL) #	1.7 (1.2–2.6)	2.0 (1.2–2.9)	0.19	2.4 (1.7–3.8)	2.4 (1.8–4.3)	0.86
Endotoxin Activity#	1.6 (1.1–2.4)	1.9 (1.2–2.7)	0.18	2.5 (1.6–4.3)	2.9 (1.7–5.0)	0.35
25(OH) D (nmol/L) #	44.2 (23.7–74.5)	55.1 (32.3–83.2)	0.06	41.3 (28.4–57.8)	40 (25.9–61.3)	0.87
TL (Kbp)	6.0 ± 1.5	5.8 ± 1.4	0.62	5.5 ± 1.3	5.6 ± 1.2	0.54

**Note:** Normal continuous data is expressed as mean ± standard deviation, non-normal continuous data (#) is presented as median (Q1, Q3) whilst categorical data (^) are presented as frequency (%). The “P-value” value < 0.05 was considered as statistically significant.

### 3.3 Bivariate associations of TL with anthropometric and biochemical parameters in the study groups

**Fig. 2:**
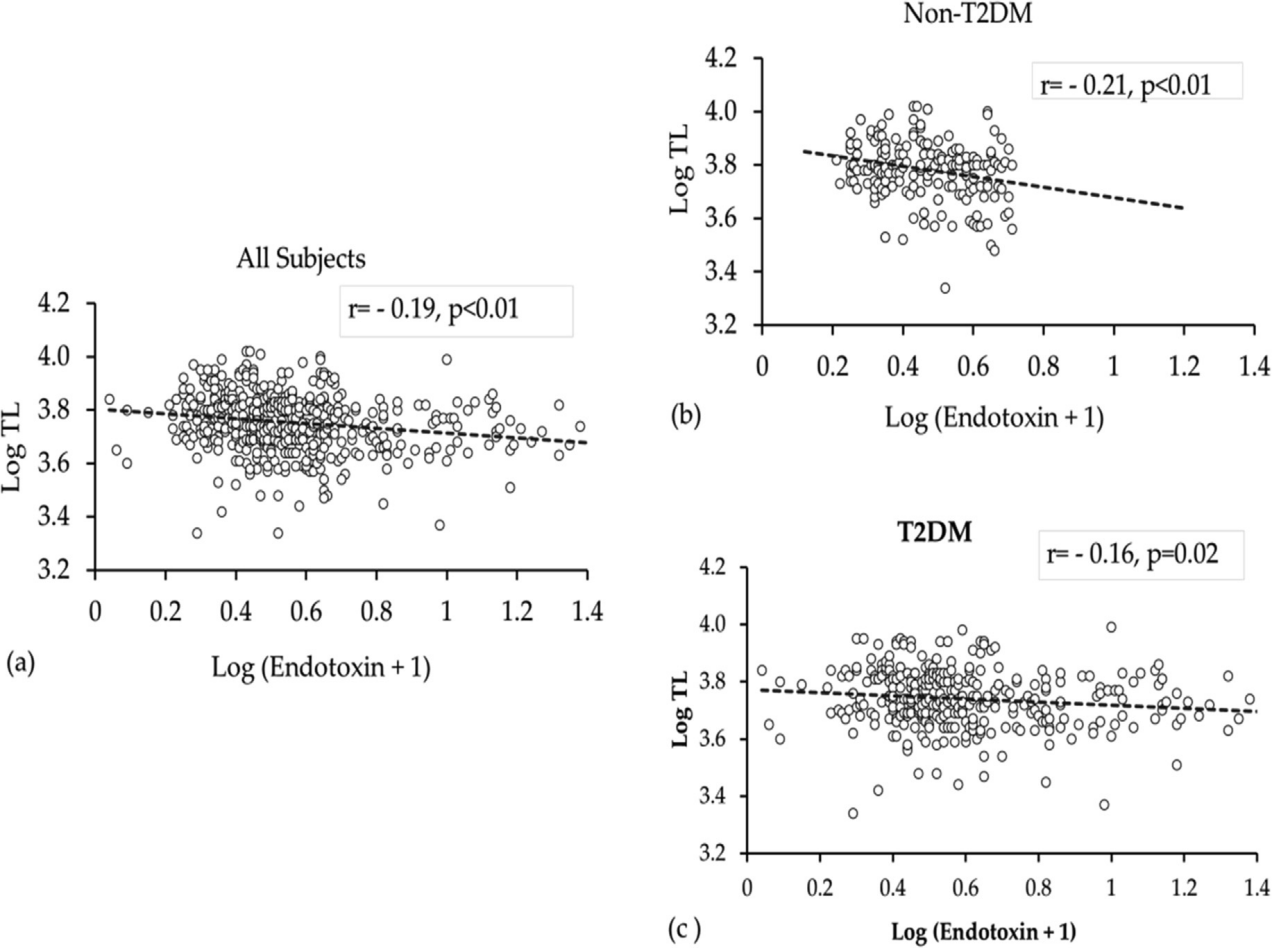
Association of normalized values of TL and log Endotoxin a) all subjects, b) non-T2DM, and c) T2DM. The trend line shows inverse correlation between log Endotoxin and TL.

Bivariate associations with TL in the study groups were analyzed (Table 3). In all non-obese subjects. TL was inversely related with LDL-cholesterol, and endotoxin activity. Significant inverse associations between TL, endotoxin and endotoxin activity were also observed in the obese group. After stratification according to T2DM status, TL showed significant inverse associations with age, total and LDL-cholesterol as well as the activity of endotoxin in the non-obese group, non-T2DM group, while no associations were noted in the non-T2DM obese group. In the non-obese T2DM group, TL was inversely associated only with triglycerides and finally in the obese T2DM group, TL was inversely associated only with HDL-LDL ratio. In all subgroups 25(OH)D was not associated with neither TL nor endotoxin. The bivariate associations of TL with endotoxin in all subjects and according to DM status were noted (Fig. 2).

**Table 3:** Bivariate Associations of TL with Clinical Characteristics of Groups.

**Parameters**	**Overall** **(n = 775)**		**Non-T2DM (n = 388)**		**T2DM** **(n = 387)**	
	**Non-Obese**	**Obese**	**Non-Obese**	**Obese**	**Non-Obese**	**Obese**
Age (years)	0.00	−0.05	0.25**	−0.01	−0.11	−0.07
BMI (Kg/m^2^)	0.04	0.01	0.00	−0.01	0.08	0.04
Waist (cm)	−0.03	−0.05	0.01	−0.04	0.00	−0.06
Hips (cm)	0.00	0.05	0.00	0.06	0.02	0.04
Systolic BP (mmHg)	−0.04	0.02	0.07	0.12	−0.08	−0.08
Diastolic BP (mmHg)	−0.02	−0.07	−0.01	−0.04	−0.02	−0.10
Total Cholesterol (mmol/L)	−0.09	0.01	−0.25**	0.11	0.05	−0.07
HDL Cholesterol (mmol/L)	−0.03	0.05	−0.02	0.04	−0.03	0.06
LDL Cholesterol (mmol/L)	-0.013*	0.00	−0.23**	0.12	−0.02	−0.11
LDL-HDL ratio	−0.09	−0.06	−0.14	−0.01	−0.04	−0.18*
Triglycerides (mmol/L) #	0.08	0.00	−0.04	0.00	0.20**	0.02
Insulin (μU/mL) #	0.05	−0.02	0.01	−0.08	0.09	0.05
HOMA-IR#	0.07	−0.03	0.05	−0.09	0.18*	0.09
HOMA-β#	0.09	0.00	0.03	−0.04	−0.07	−0.04
25(OH) D (nmol/L) #	0.02	−0.03	−0.01	−0.04	0.04	−0.06
Endotoxin (EU/mL)	-0.019**	−0.17**	−0.26*	−0.20	−0.10	−0.13
Endotoxin activity#	−0.17*	−0.16*	−0.28*	−0.16	−0.06	−0.13

**Note:** Data is presented as coefficient (r); ** denotes p-value < 0.01 and * denotes p-value < 0.05.

### 3.4 Significant predictors of TL, endotoxin and 25(OH)D

Using TL as the dependent factor and all other parameters as independent factors the current data showed that endotoxin was consistently a significant predictor for TL in all subjects, even after stratification according to subgroups, with 9–20% of the variance in circulating TL influenced by endotoxin (Table 4). Using endotoxin as dependent variable data analysis showed that TL was also the single most significant predictor in the obese T2DM group as well as the non-obese, non-T2DM groups, with other groups showing other cardiometabolic parameters together with TL. Lastly, no significant predictors were elicited in the T2DM group alone using 25(OH)D as dependent factor, with glucose and HDL-cholesterol predicting 8% of the variances in 25(OH)D in all subjects. In the non-T2DM group however, TL and HDL together predicted 29% of the variances obtained in 25(OH)D levels. Only HDL-cholesterol was the significant predictor for 25(OH)D in the obese, non-T2DM group.

**Table 4:** Significant Predictors of TL, Endotoxin and 25(OH)D.

**Group**	**N**	**Dependent Variables**		
		**TL**	**Endotoxin**	**25(OH)D**
All	775	Endotoxin adj. R^2^ = 0.09; p < 0.001	TL, Triglycerides, Total Cholesterol adj. R^2^ = 0.16; p < 0.001	Glucose, HDL adj. R^2^ = 0.08; p < 0.001
**T2DM Group**				
Obese	198	Endotoxin adj. R^2^ = 0.04; p = 0.03	TL adj. R2 = 0.05; p < 0.001	–
Non-Obese	181	Endotoxin, Glucose, Triglyceride s adj. R^2^ = 0.20; p < 0.001	TL, Triglycerides, Total Cholesterol adj. R^2^ = 0.21; p = 0.02	–
**Non-T2DM Control Group**				
Obese	197	Endotoxin adj. R^2^ = 0.10; p = 0.003	TL, Triglycerides, Total Cholesterol adj. R^2^ = 0.19; p < 0.001	HDL adj. R^2^ = 0.04; p = 0.046
Non-Obese	171	Endotoxin adj. R^2^ = 0.12; p = 0.007	TL adj. R^2^ = 0.09; p = 0.015	TL, HDL adj. R^2^ = 0.29; p < 0.001

**Note:** Independent variables entered include age, BMI, sex, glucose, insulin, triglycerides, total cholesterol, HDL- and LDL-cholesterol. TL, endotoxin and 25(OH)D were included in the model unless they are dependent variables.

## 4 Discussion

This present study represents one of the first studies to demonstrate the significant associations of gut-derived endotoxin in accelerated biological ageing amongst adults influenced by adiposity and metabolic state. Specifically these present finding highlights that endotoxin represents a significant predictor of TL reduction to be maintained across all groups studied in this Arab cohort, from non-obese, obese through to non-T2DM and T2DM groups. Furthermore, that regression analysis with TL has showen that endotoxin predicts between 10 and 12% of the TL variance perceived amongst subjects who were non-T2DM, with and without obesity. Our studies also confirmed, in this Arab ethnic cohort, that the TL was reduced in the subjects with T2DM, coupled with raised endotoxin levels as well as an associated relationship between the two. Also, in the non-T2DM, non-obese group, TL and HDL-cholesterol predicted as much as 29% of the variances perceived in 25(OH)D, offering insights as to the potential protective function that vitamin D may offer to subjects with increased weight gain and early metabolic dysfunction.

In this current study, whilst endotoxin and TL were not significantly altered by adiposity alone, bivariate associations did reveal endotoxin with adiposity and metabolic status. The inverse significant associations between endotoxin and TL appeared strongest in the non-T2DM population, particularly amongst the non-obese, independent of other cardiometabolic risk factors. The importance of this association is reinforced by several lines of previous evidence in the current literature suggesting that circulating endotoxin is not only a biomarker of metabolic dysfunction but also a mediator in the pathogenesis of obesity mediated T2DM, with endotoxin implicated as a potential source in this case to also enhance TL attrition. Such an association may suggest a delicate interplay between the gut microbiome and ageing that is sensitive to an individual’s metabolic status (Wilmanski et al., 2021). Furthermore, enhanced adipocyte hypertrophy and hyperplasia in obesity leads to exacerbated infiltration by inflammatory cells resulting in increased oxidative and endoplasmic reticulum (ER) stress (Fernández-Sánchez et al., 2011) in addition to cellular telomeric damage by the T2DM state and hence accelerated TL shortening amongst individuals who were obese with T2DM. Nevertheless, the exact mechanism through which endotoxemia may influence TL attrition may not be known at present; however, increased oxidative stress during endotoxemia may be a link to this association as an inverse correlation between TL and oxidative stress has been suggested before (Demissie et al., 2006, Gavia-García et al., 2021). Oxidative stress *in-vivo* is known to be accompanied by an increased expression of enzymes that exert anti-oxidant effects as a part of cellular stress response pathway (Kourtis and Tavernarakis, 2011). The imbalance in this cellular stress response mechanism, as exacerbated in age-related chronic disorders such as T2DM, is one of the contributing factors in systemic inflammation (-Victor et al., 2011)]. The current findings in this study also highlight that the rate of TL shortening with endotoxin appeared stronger in the non-T2DM than the T2DM group. As such this may suggest that other regulatory mechanisms apart from imbalance in oxidative stress/anti-oxidative response may contribute towards this inverse relationship between TL and endotoxin. Importantly as well, these studies show that irrespective of adiposity and insulin resistance status a continuous associative relationship endotoxin with TL remains which indicates its potential use as a biomarker of progressive biological ageing. In addition as endotoxin may serve as a marker for biological ageing this biomarker could be utilized as an indicator of premature ageing and health (Adams and White, 2004).

As expected, the circulating levels of endotoxin in T2DM subjects in this study was significantly elevated compared with non-T2DM, independent of sex, confirming our previous observations in Arab and other ethnicities (Al-Attas et al., 2009, Harte et al., 2012, Al-Disi et al., 2015, Al-Disi et al., 2020). Furthermore a prior systematic review of 14 studies with 9773 subjects with T2DM highlighted that 66.4% of subjects had raised circulating endotoxin compared with non-T2DM subjects (Gomes et al., 2017) which corroborates with the findings in this study. The elevation in circulating in endotoxin indicate that the increased intestinal permeability in T2DM favors translocation of endotoxin across the gut intestinal epithelium leading to elevated levels (De Kort et al., 2011). Once in circulation, the lipid A component of endotoxinis recognized by MD2-TLR4 receptors of innate immune system initiating the cascade of biosynthesis of inflammatory cytokines, via activation of nuclear factor κB (NF-κB) (Wang et al., 2017). Moreover, insulin resistance noted in T2DM has been suggested to impair the functioning of neutrophils, macrophages and other monocytes which are important in clearing the bacterial products from the circulation leading to further raised endotoxin concentrations (Torres-Castro et al., 2016). While causality is out of the present study’s scope, the observations mentioned previously highlight that chronic inflammation, which is a hallmark of ageing, can be associated with gut dysbiosis (Buford, 2017), which can be potential sources of novel treatments in the future to combat premature biological ageing.

Lastly, the present study observed that TL, together with HDL, are significant predictors of 25(OH)D at least in the non-obese, non-T2DM individuals as this association was not observed in other groups. While the exact mechanism remains unclear for this, there is a growing interest on the influence of vitamin D in premature ageing due to its important role in vital cellular processes including apoptosis (Kord-Varkaneh et al., 2020), how ageing itself alters vitamin D metabolism (de Jongh et al., 2017) as well as the inverse association between vitamin D status and most age-related diseases including mortality (Zarei et al., 2020).

The authors acknowledge some limitations. The causal relationship between the variables in question cannot be established because of the observational nature of the study. Furthermore, as TL biology is complex, several important associated factors, including, physical activity and diet were not examined in this study. Other markers of gut permeability such as intestinal fatty-acid binding protein I-FABP and soluble CD14 (sCD14) as well as inflammatory cytokines which may likely influence TL attrition were also not assessed. Despite these limitations, the findings have merit as it demonstrates for the first time the influence of gut-derived endotoxin and vitamin D in premature ageing, in a cohort of adult Arabs with varying levels of insulin resistance and adiposity.

## 5 Conclusions

The present findings indicate that circulating endotoxin is associated with TL attrition independent of obesity or T2DM status, highlighting the potential role of endotoxin as a marker for biological ageing. The significant association of vitamin D with TL in the non-obese, non-T2DM population also merits further investigation, taking into consideration other markers of gut permeability and inflammation. Taken together, intervention studies that aim to reverse or mitigate premature biological ageing through gut microbiome manipulation influencing systemic endotoxin levels or vitamin D correction may confirm our present findings.

Institutional Review Board Statement.

The study was approved by the Ethics Committee of College of Science, King Saud University (KSU), Riyadh Saudi Arabia (IRB no.8-25-454239). The experiments conducted in this study were done according to the ethical standards of the committee responsible for human experimentation (institutional and national) which are in accordance with the guidelines set in 1975 and revised in 2013 by declaration of Helsinki.

Informed Consent Statement.

Informed consent was obtained from all subjects involved in the study.

## Author Contributions

Study Design: N.M.A.-D. and P.G.M; Subject selection and data collection: S.A.; Sample analysis: M.G.A.A.; Manuscript draft preparation: S.S.; Data interpretation: S.S.; P.G.M.; and S.A.; Manuscript review: P.G.M., G.T., G.P.C., S.S. and N.M.A.-D; Project supervision: N.M.A.-D and P.G.M.

## Declaration of Competing Interest

The authors declare that they have no known competing financial interests or personal relationships that could have appeared to influence the work reported in this paper.
